# Screening and brief intervention for alcohol use in a surgical oncology unit: framework, educational program, and qualitative analysis of the implementation process

**DOI:** 10.1186/1940-0640-7-S1-A20

**Published:** 2012-10-09

**Authors:** Marion Barrault, Marianne Saint-Jacques, Gilliard Jérôme, Grados Clarisse, Garguil Véronique, Anne Bouyer, Lakdja Fabrice, Bussieres Emmanuel

**Affiliations:** 1 Department of Psychology, Bergonié Institute, Bordeaux, France; 2 Department of Addiction Psychology, Saint Nicolas Hospital Center, Blaye, France; 3 Department of Addiction Treatment, Charles Hospital Center, Bordeaux, France

## 

It is well-documented that alcohol and tobacco consumption reduce treatment efficacy, increase side effects, may encourage relapse and/or secondary cancers, and affect quality of life in patients being treated for cancer. Because it represents a “teachable moment” for many of those diagnosed, cancer may create a window of opportunity in which to intervene, to help patients identify behaviors that put them at risk for poor health outcomes, and to provide them with the support required to make and adhere to desired lifestyle modifications. Despite scientific, clinical, and political incentives, SBI for risky alcohol use in cancer settings is poorly implemented. Stigmatization of people with addictive behaviors and lack of education about the health consequences of at-risk alcohol use may contribute to the limited dissemination of SBI. We present the framework of implementation for a clinical program of screening, case finding, and brief intervention for first-time cancer patients with at-risk alcohol behavior in a surgical oncology unit. Our educational program for health caregivers focuses on screening and detection procedures, interpersonal skills, openness to implementing new intervention programs, types of interventions, and SBI training. We also present results of a preliminary qualitative analysis of human and organizational barriers and facilitators to implementing an SBI program. Implementing specific training courses may help health professionals placing the problem of alcohol in a new perspective and eliminate the aspects of fatalism and resignation which are so often observed.

